# An environmental green factorial design/assisted spectrofluorimetric technique for quantitation of citicoline in pharmaceutical dosage form and wastewater

**DOI:** 10.1038/s41598-025-19263-5

**Published:** 2025-09-15

**Authors:** Maha Mahmoud Abou El-Alamin, Ola Abd Elkhalek, Marwa M. Azab

**Affiliations:** Department of Pharmaceutical Analytical Chemistry, Faculty of Pharmacy, Helwan University, P.O.Box 11795, Cairo, Egypt

**Keywords:** Citicoline, Spectrofluorimetry, Derivatization, Green assessment, Wastewater, Chemistry, Environmental sciences

## Abstract

A novel, sensitive, and straightforward spectrofluorimetric method was developed for the quantitative determination of citicoline in pharmaceutical formulations and wastewater. The technique depends on the efficient derivatisation of the primary amino group of citicoline with O-phthalaldehyde (OPA) and N-acetylcysteine (NAC) in borate buffer (pH 11), yielding a highly fluorescent derivative. The fluorescence intensity was measured at 425 nm with an excitation wavelength of 341 nm. Experimental parameters affecting the derivatisation reaction were thoroughly optimised. Under optimal conditions, the method exhibited a linear response over the concentration range of 50.0–300.0 ng/mL, with an excellent correlation coefficient (R^2^ = 0.9942). The limits of detection (LOD) and quantification (LOQ) were 6.4 ng/mL and 19.5 ng/mL, respectively. The approach demonstrated high accuracy and precision, with per cent recovery values close to 100% and a relative standard deviation (RSD) of 0.512%, confirming its reliability. Validation was done in agreement with ICH Q2(R1) guidelines, and statistical comparison with previously reported approaches indicated no significant difference in performance. The sustainability of the method was studied using the analytical greenness tools confirms the method’s eco-friendly profile. Collectively, these contributions advance drug bioavailability while promoting a more efficient and sustainable analytical framework.

## Introduction

**Fig. 1:**
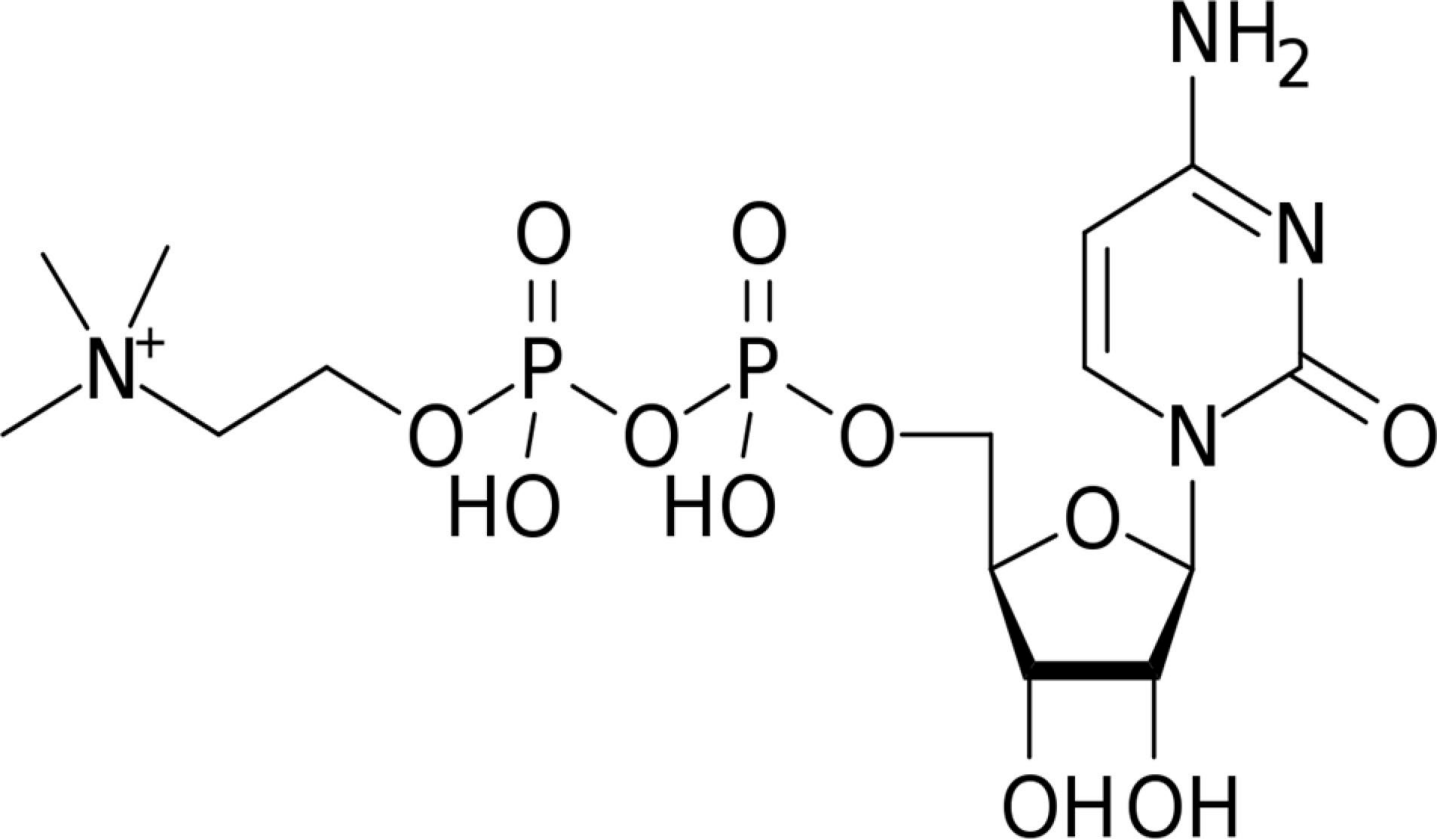
Chemical structure of CTN.

Citicoline (CTN) is a naturally occurring endogenous compound that plays a significant role in the biosynthesis of phospholipids, which are important components of cell membranes, particularly in neuronal tissues. Due to its neuroprotective and neurorestorative properties, CTN has been extensively studied for the treatment of various neurological problems. It has demonstrated therapeutic potential in the management of intracerebral haemorrhage, a condition associated with a worse prognosis than cerebral infarction^1^. Additionally, CTN has been presented to decrease mortality in experimental models of subarachnoid haemorrhage and arterial occlusion^2–4^. Beyond its cerebrovascular benefits, CTN has been reported to enhance cerebral glucose metabolism and increase cerebral blood flow, further supporting its role in brain function and recovery^5^. CTN is chemically known as cytidine 5’-diphosphocholine, as shown in Fig. 1.

Many analytical methods have been explored for the quantification of CTN in different pharmaceutical dosage forms. Spectrophotometric approaches are widely used due to their simplicity and accessibility. For instance, Surani et al. developed a UV-visible method with a linearity range of 5–55 µg/mL (*r* = 0.9983)^6^Pathan et al. reported linearity in the range 5–15 µg/mL using a difference spectroscopic technique^7^Sivadas et al. established linearity for citicoline between 5 and 13 µg/mL^8^. Many other Spectrophotometric approaches are also used to quantify CTN^9–12^Additionally, potentiometric methods offer higher sensitivity with a linearity range from 1.00 × 10^− 8^ to 1.00 × 10⁻² M^13^. Chromatographic techniques, including various RP-HPLC methods, offer improved specificity and broader linearity ranges. Maradiya and Pansara reported linearity between 10 and 60 µg/mL^14^while Bindaiya & Argal achieved linearity over 20–100 µg/mL (r² = 0.9999)^15^An Ecologically Sound RP-HPLC Method for Estimating Citicoline using AQbD with Degradation Studies demonstrated linearity in the 10–50 µg/mL range (r² ≈ 0.999)^16^and other validated RP-HPLC approaches extended the linear range up to 80–180 µg/mL (r² = 0.9999)^17^. Additionally, an RP-HPLC method using an internal standard achieved linearity in the range 5–25 µg/mL^18^. High-performance liquid chromatography (HPLC) and other chromatographic techniques have been developed to offer higher specificity and separation efficiency^19–24^. However, despite their widespread use, these conventional approaches have notable limitations. They often involve high costs, lengthy procedures, extensive sample preparation, and the utilization of large quantities of organic solvents.

Only one spectrofluorimetric technique, depending on the quenching of eosin dye, has been developed^25^ This method demonstrated linearity over the range of 300–3000 ng/mL (0.3–3.0 µg/mL) with R² of 0.9996, and LOQ and LOD values of 291.0 ng/mL and 93.86 ng/mL, respectively. However, this method has several limitations. Many compounds, especially those found in complex biological or pharmaceutical samples, can also quench eosin fluorescence, leading to false positives or inaccurate results^26,27^. Additionally, Eosin is also susceptible to photobleaching under prolonged light exposure, which can reduce fluorescence intensity over time and affect reproducibility^28^. At higher concentrations of eosin, self-quenching can occur, diminishing fluorescence and limiting the linear detection range^29^.

These challenges highlight the need for a more reliable and sensitive spectrofluorimetric approach for CTN determination. Spectrofluorimetric methods are particularly advantageous due to their inherently high sensitivity, low detection limits, wide applicability for trace-level analysis, and minimal sample preparation requirements. Moreover, they often consume smaller amounts of solvents and reagents compared to chromatographic methods, making them more cost-effective and environmentally friendly

## Experimental

### Regents and materials

- CTN pure sample (98.9% according to the certificate from the company) was kindly taken from the October pharma company. Fortamind^®^ (Global Pharmaceutical Industries) 100 mg/mL Oral drops (batch number 2278004) were obtained from the local market.
- O-Phthalaldehyde (OPA) was taken from SD Fine Chemicals (Mumbai, India). N-acetylcysteine (N-AC, 99%), cysteine, and acetylacetone (98%) were purchased from Loba Chemie (Mumbai, India). Formaldehyde solution (36%) was also procured and used as received in the experimental procedures. Ninhydrin and phenylacetaldehyde were taken from Sigma-Aldrich Chemie GmbH, Steinheim, Germany.
- Methanol, glacial acetic acid, acetonitrile, mercaptoethanol (ME), and ethyl acetoacetate were obtained from Sigma Aldrich, Germany.
- Boric acid, potassium chloride, sodium chloride, citric acid, phosphoric acid, hydrochloric acid, potassium dihydrogen phosphate, and mercaptoethanol were received from El-Nasr Chemicals, Cairo, Egypt.
- Double-distilled water was utilised for the preparation of all solutions.

### Instrumental and software

- Spectrofluorometric measurements were performed utilising a JASCO FP-6200 spectrofluorometer equipped with 1.0 cm quartz cells and a 150 W xenon lamp as the excitation source. Both the excitation and emission slit widths were optimised to 10 nm. Spectral data acquisition and processing were performed using The Spectra Manager™ Suite (FP-6200 Control Driver Software, Version 1.54.03, JASCO Corporation, Tokyo, Japan), which was employed to control the spectrofluorometer and acquire data. Further information is available at: https://jascoinc.com.
- pH adjustments of buffer solutions were conducted utilising a digital pH meter (Hanna Instruments, Model HI-2211, USA).
- Sample preparation involved the use of a bench-top ultrasonic bath (POWERSONIC410, USA).
- a digital analytical balance (Denver Instrument, Model TP-214).
- A tabletop low-speed centrifuge (Cence Group Hunan Xiang Yi Laboratory Instrument Development Model L600).
- A free trial version of Minitab^®^ Statistical Software (Version 21.4.1, Minitab LLC, State College, PA, USA) was utilised to perform the statistical analysis of the factorial design data. Further information is available at: https://www.minitab.com.

### Preparation of a standard solution

A standard CTN stock solution (0.5 mg/mL) was prepared by dissolving 5.0 mg of CTN in distilled water and diluting to 10.0 mL in a volumetric flask. From this, a working standard solution with a concentration of 5.0 µg/mL was prepared by diluting 100 µL of the stock solution to 10.0 mL with the same solvent. All solutions were stored in dark glass containers at 4 °C and remained stable for at least seven days.

To prepare a borate buffer at pH 11, equal volumes of 0.2 M sodium hydroxide and 0.2 M boric acid were mixed, and the final pH was fine-tuned by utilising 0.2 M NaOH. An acetate buffer (pH 4) was obtained by adding appropriate volumes of 0.2 M sodium acetate and 0.2 M acetic acid^30^. The Torell and Stenhagen buffer (pH 8.2) was prepared by mixing equal volumes of 1 M citric acid, 1 M phosphoric acid, and 1 M sodium hydroxide, and adjusting the pH with 0.1 M hydrochloric acid^31^.

Formaldehyde and acetylacetone (AAF) reagents were freshly made each day by combining 300 µL acetylacetone and 700 µL formaldehyde with 3.5 mL of a 5 mM sodium acetate solution and then diluting the mixture to 10.0 mL with deionised water^32^. A 0.1% w/v ninhydrin solution was freshly prepared in distilled water for each use. Phenylacetaldehyde was prepared as a 0.01% v/v ethanol solution, which remained stable for up to a week when stored at 4 °C^33^. Both O-phthalaldehyde (OPA) and N-acetylcysteine (N-AC) solutions were freshly prepared at 1 mg/mL by dissolving 25.0 mg of each compound in 25-mL volumetric flasks. For OPA, the reagent was first dissolved in 5.0 mL of methanol before being brought to volume with deionised water, while N-AC was directly dissolved in deionised water without methanol^32^.

### Calibration

Varying volumes (100–600 µL) of the CTN working standard solution were precisely transferred into a series of 10-mL volumetric flasks, each containing 4 mL of borate buffer at pH 11. To each flask, 600 µL of O-phthalaldehyde (1 mg/mL) and 1000 µL of N-acetylcysteine (1 mg/mL) were added, followed by thorough mixing. After allowing the reaction to proceed for 30 min, the solutions were diluted to volume with double-distilled water, yielding final CTN concentrations ranging from 50.0 to 300.0 ng/mL. A blank sample was prepared in parallel. The relative fluorescence intensity (RFI) was recorded at an emission wavelength of 425 nm following excitation at 341 nm. A calibration curve was constructed by plotting RFI against CTN concentration, and the regression equation was derived from this linear relationship.

### Preparation of a pharmaceutical dosage form

An exact volume of 1 mL from the Fortamind^®^ oral drop solution (100 mg/mL) was transferred into a 100 mL volumetric flask. A small quantity of distilled water was added, and the mixture was shaken thoroughly. The solution was then diluted to the mark with distilled water to obtain a 1.0 mg/mL solution. From this, 1 µL was transferred into a 10 mL volumetric flask and diluted to volume with distilled water. Serial dilutions were subsequently prepared to achieve concentrations of 50, 100, and 150 ng/mL. The general analytical technique was employed to determine the CTN concentration in the Fortamind oral drop solution (100 mg/mL), based on the corresponding regression equation, with three replicate analyses performed.

### Preparation of synthetic wastewater

Synthetic wastewater was prepared based on the procedure described in the referenced studies^34–36^. The formulation included saccharose and starch as organic carbon sources, along with inorganic salts such as sodium chloride, magnesium chloride, calcium chloride, and potassium phosphate to simulate the ionic composition typically found in real wastewater.

### Factorial design

Prior to designing the experiments, it was essential to conduct preliminary screening studies to identify the experimental factors that significantly influence the fluorescence intensity of the reaction products in the analytical approach. Based on the initial studies, the most influential quantitative factors were determined to be the buffer volume, ranging from 3.5 to 4 mL, N-acetylcysteine N-AC volume 900 to 1000 µL, and OPA volume 500 to 600 µL.

## Result and discussion

**Scheme 1:**
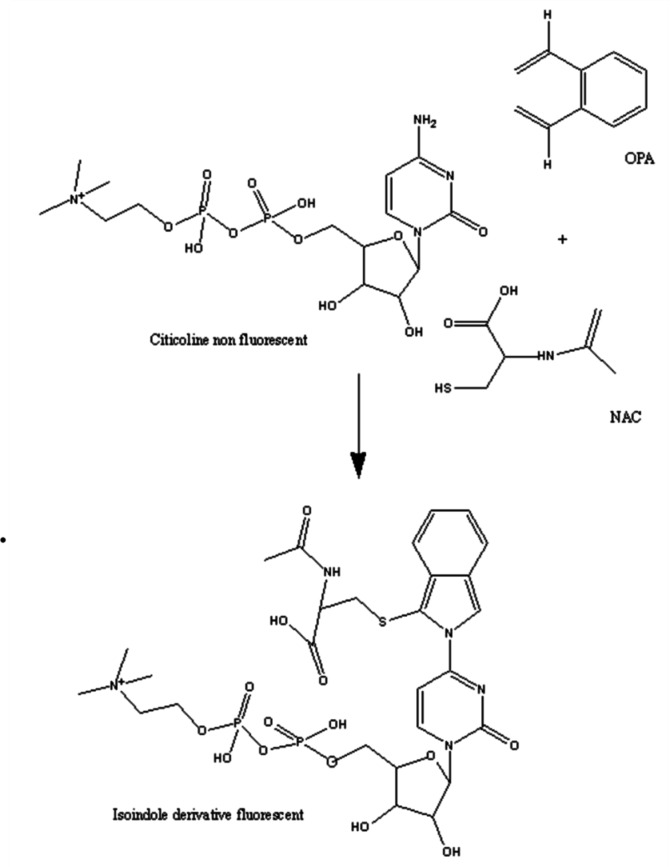
Proposed reaction mechanism between CTN and OPA/N-AC.

The lack of intrinsic fluorescence of CTN makes its detection using spectrofluorimetric techniques particularly difficult. This challenge becomes even more significant when a straightforward, sensitive, and cost-effective method like fluorescence spectroscopy is required, especially in pharmaceutical quality control laboratories. Nevertheless, the presence of a primary amine group in the molecule, which can undergo derivatisation via condensation reactions with OPA and N-AC, forming a highly fluorescent isoindole derivative, as illustrated in Scheme 1, served as the starting point for this study.

### Different derivatising agents

**Fig. 2:**
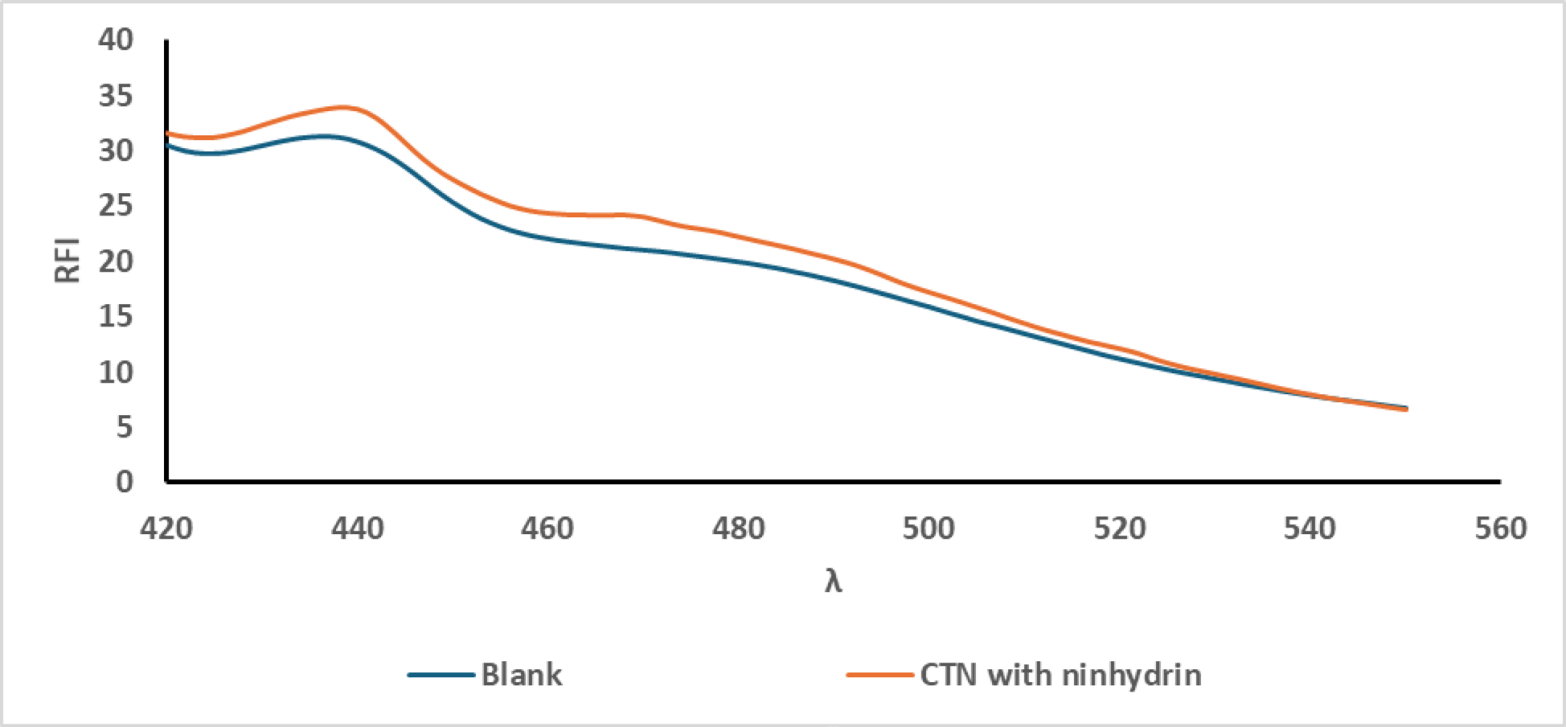
Fluorescence emission spectra of the reaction product of CTN (1.0 µg /mL) with ninhydrin after excitation at 385 nm.

**Fig. 3:**
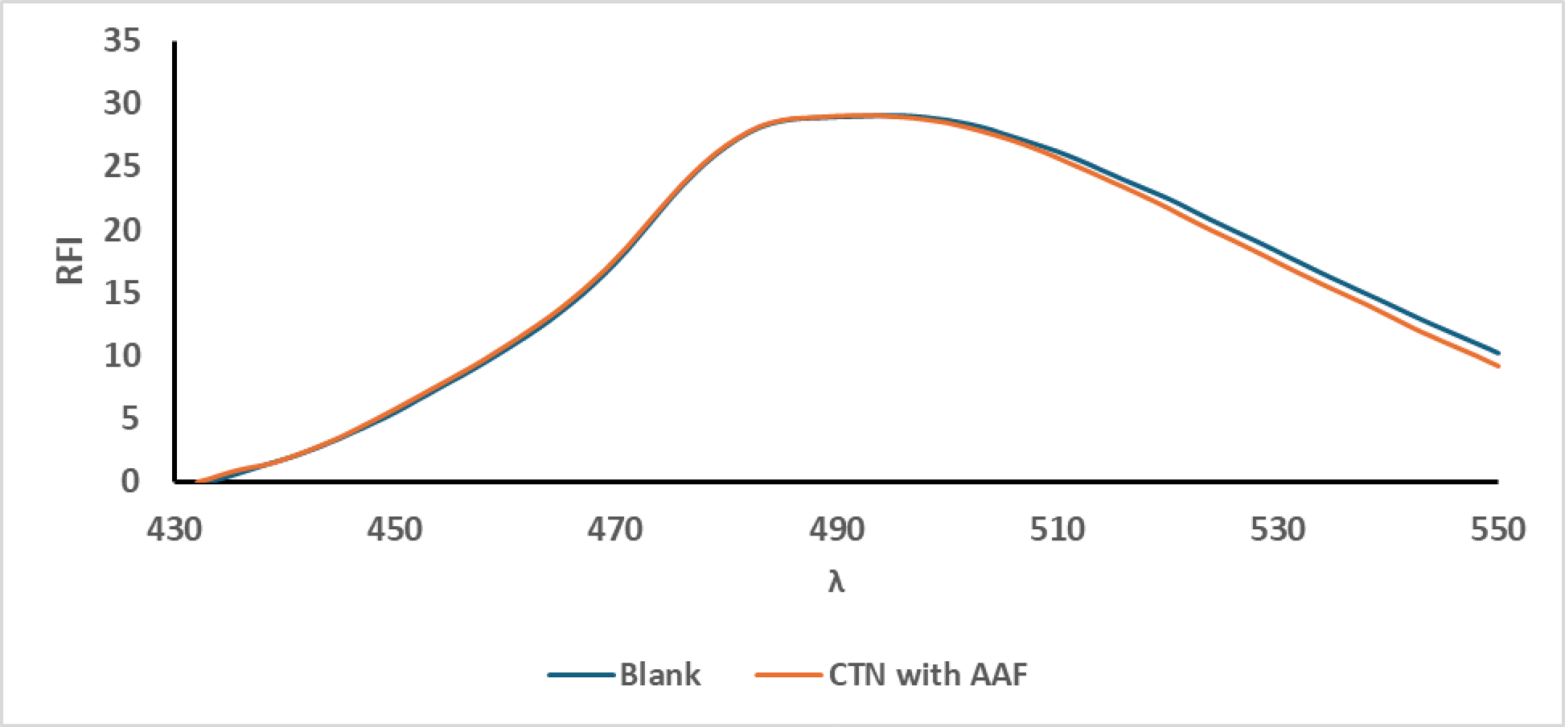
Fluorescence emission spectra of the reaction product of CTN (1.0 µg/mL) with AAF after excitation at 415 nm,

**Fig. 4:**
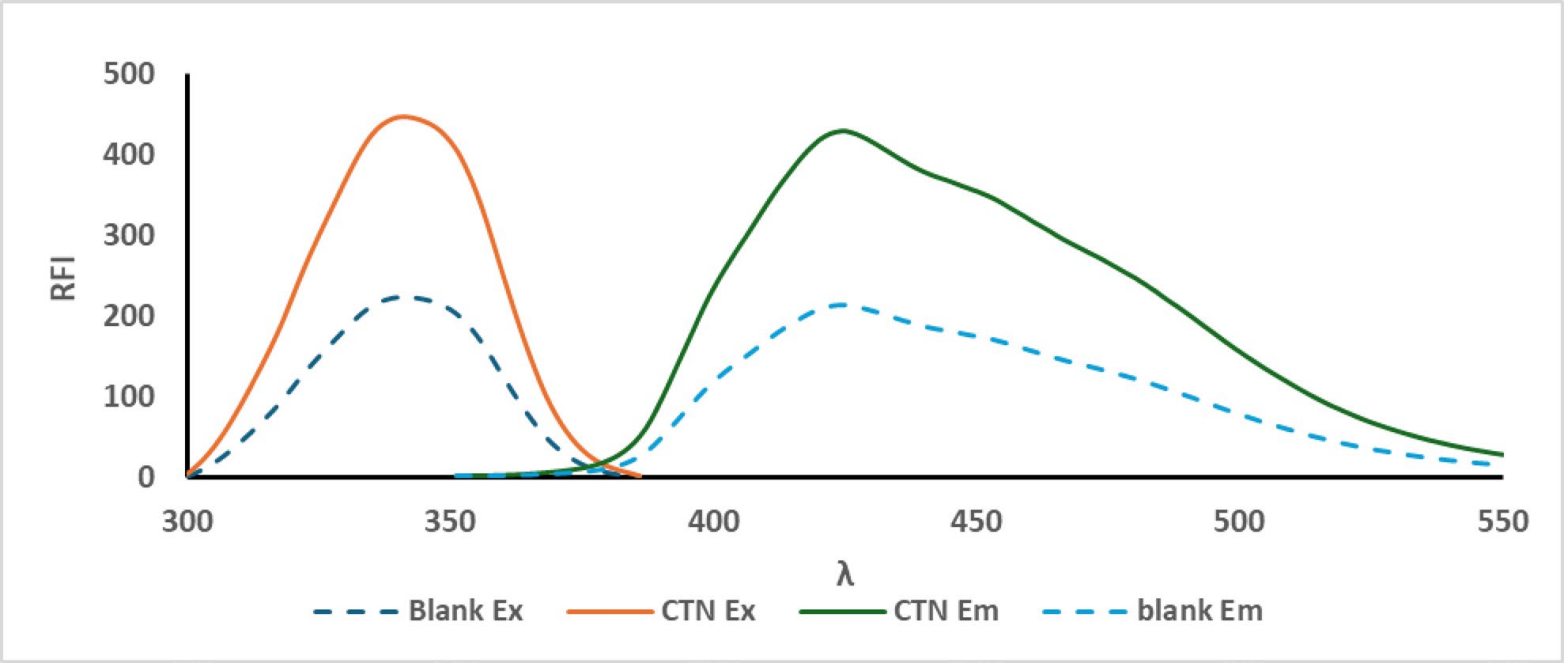
Excitation and emission spectra of the reaction products of CTN (100.0 ng/ mL) with O-PA/N-AC.

Selecting an appropriate derivatisation approach involves careful evaluation of various factors such as environmental impact, sensitivity, cost-effectiveness, the required reaction conditions, and chemical reactivity. In this study, several derivatising agents were assessed, as OPA demonstrated efficient reactivity with CTN in the presence of a thiol compound, which will be discussed in detail later. This makes OPA a cost-effective option, enabling reduced analysis expenses while maintaining high analytical performance. In contrast, both ninhydrin and the Hantzsch condensation method with AAF failed to react with CTN, as illustrated in Figs. 2 and 3, respectively, and were thus considered unsuitable for its derivatisation. Conversely, OPA enabled highly sensitive detection, confirming its suitability for this application as presented in Fig. 4.

### Different thiol group

**Fig. 5:**
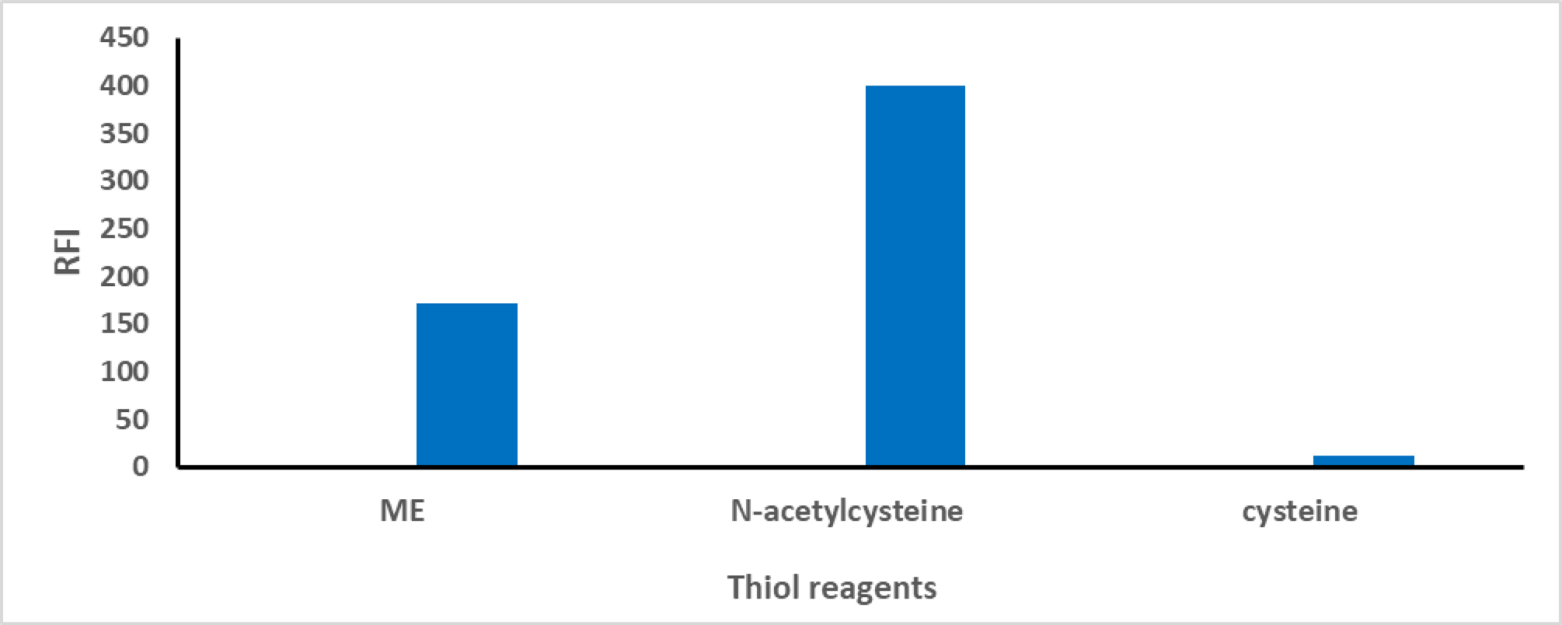
The influence of various thiol reagents on the RFI of the reaction product formed between CTN (100.0 ng/mL) and OPA.

The derivatisation of CTN using (OPA) in the presence of a thiol reagent was explored as an essential condition for its spectrofluorimetric analysis. To improve safety, several thiol reagents were evaluated as alternatives to mercaptoethanol (ME), which is associated with high toxicity and a strong, unpleasant odour^32^. The reagents examined included ME, N-acetylcysteine (N-AC), and cysteine. As shown in Fig. 5, cysteine was ineffective in stabilising the formed derivative, resulting in weak fluorescence signals. In contrast, using N-AC led to a marked improvement in RFI compared to ME due to its low toxicity and safety at high concentrations. N-AC was selected as the preferred thiol reagent^37^.

### Derivatising agent (OPA/N-AC) volume

Many preliminary experiments were performed to evaluate the influence of N-AC 1 mg/mL volume on RFI of the isoindole derivative while keeping the volume of OPA constant, and vice versa. The tested volume ranges were 100–1000 µL for OPA and 200–1400 µL for N-AC. Volumes between 500 and 600 µL for OPA and 900–1000 µL for N-AC were found to be sufficient to ensure complete condensation between CTN and OPA/N-AC. Beyond these volumes, a gradual decline in RFI was observed, attributed to isoindole destabilisation caused by excess OPA, leading to the formation of a non-fluorescent product^38^and elevated blank signals caused by excessive N-AC, both contributing to reduced fluorescence intensity. Therefore, the volumes of OPA and N-AC were subsequently optimised using the Quality by Design (QbD) approach.

### Buffer pH

**Fig. 6:**
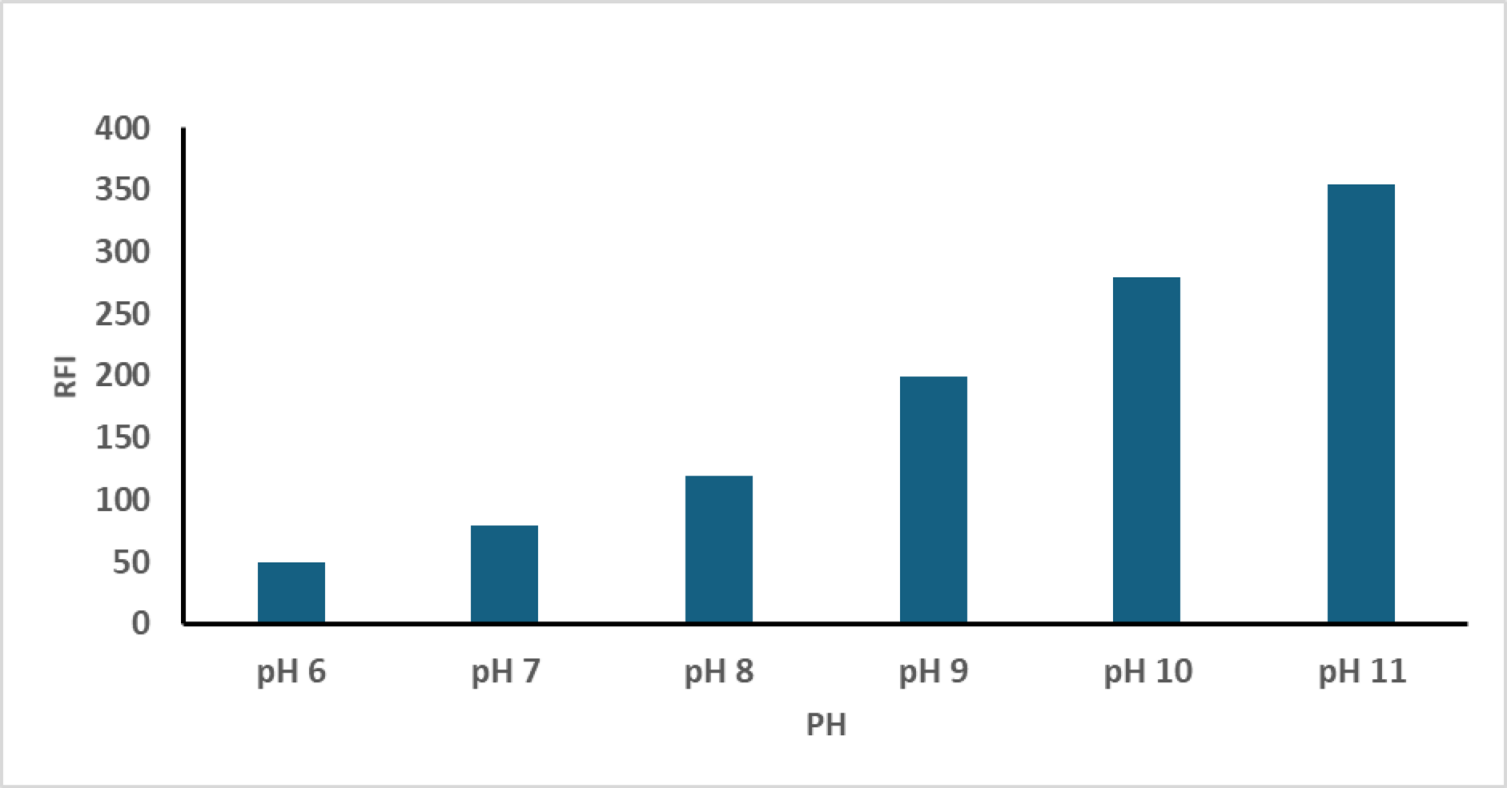
Effect of buffer pH on the RFI of the reaction product of CTN (100.0 ng /mL) with OPA/N-AC.

Initial studies confirmed that the RFI of the resulting derivative was significantly affected by pH. To assess this effect, phosphate buffer (pH 6–8) and borate buffer (pH 8–11) were employed. The maximum RFI was recorded at pH 11, indicating it as the optimal condition for the reaction, as shown in Fig. 6.

### Volume of buffer

The influence of buffer volume was evaluated within the range of 0.5 to 5.0 mL, with the optimal volume selected between 3.5 and 4.0 mL; consequently, volumes of 3.5 and 4.0 mL were further assessed using a QbD approach.

### Diluting solvent

**Fig. 7:**
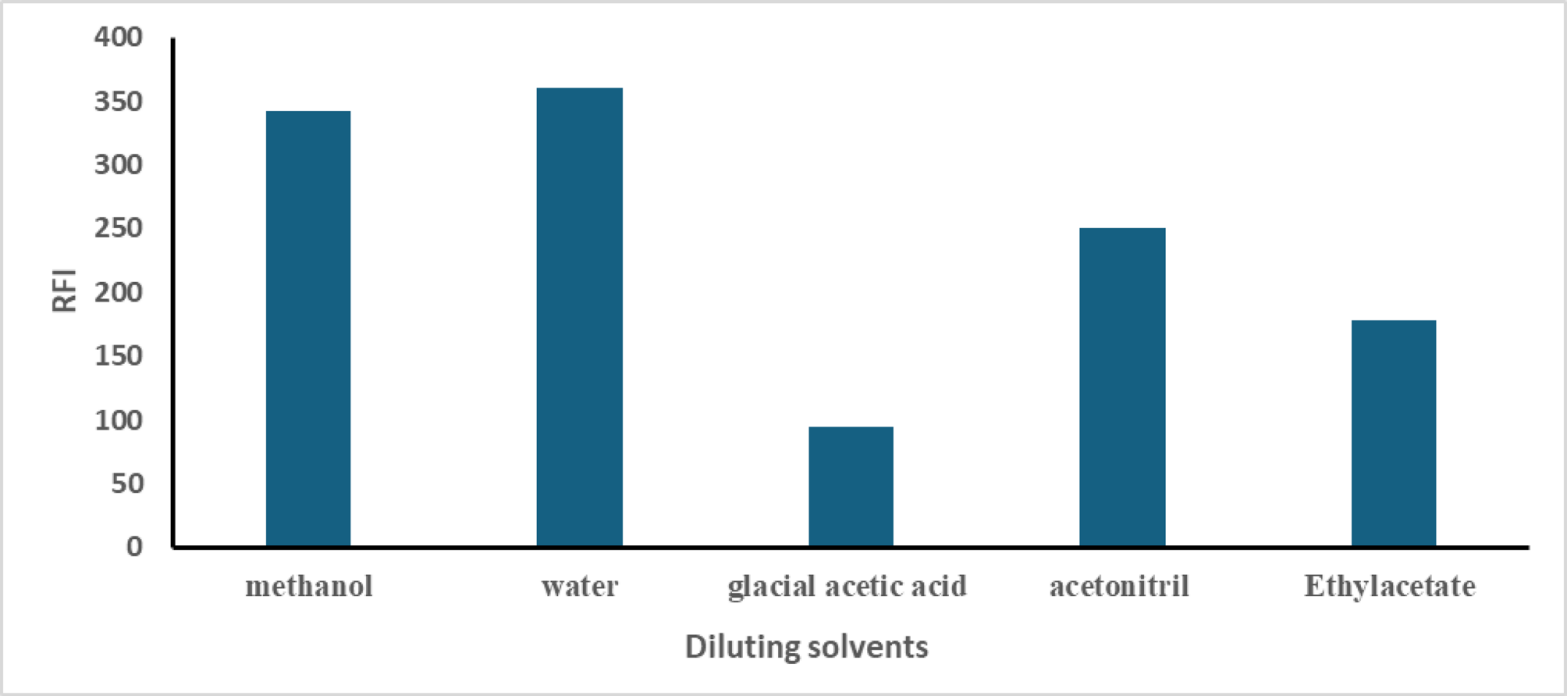
Influence of diluting solvent on RFI of CTN 100 ng/ml with OPA/NAC.

Different solvents, including water, methanol, ethyl acetate, glacial acetic acid, and acetonitrile, were examined for their suitability as diluents for the reaction product, as shown in Fig. 7. The highest fluorescence intensities were observed with water and methanol. Due to its lower cost, non-toxicity, and greener profile, water was selected as the preferred diluting solvent over methanol.

### Reaction time

**Fig. 8:**
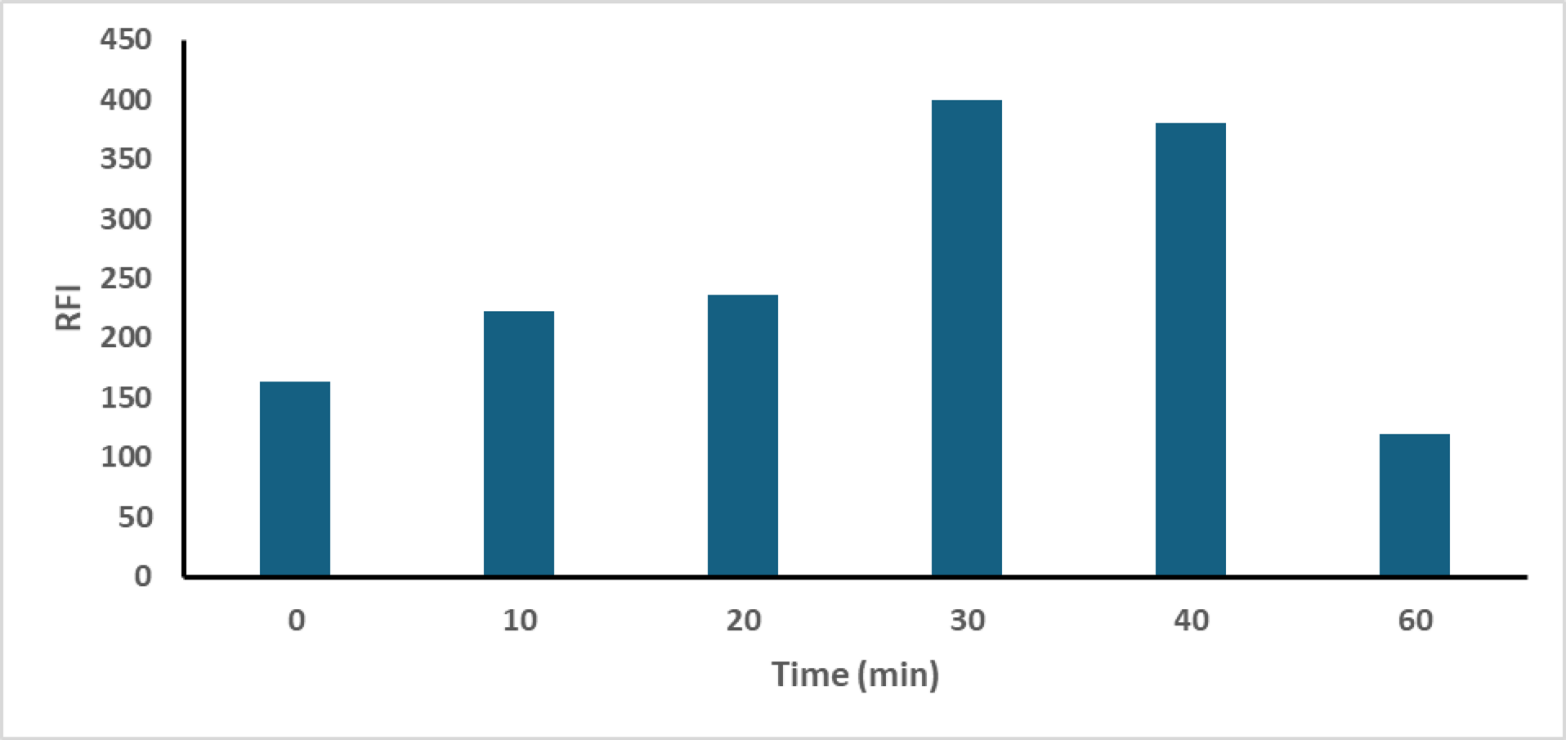
Influence of reaction time on RFI of reaction product of CTN (100.0 ng/ml) with OPA/N-AC.

The influence of reaction time was studied by conducting the reaction between CTN and OPA/N-AC over intervals ranging from 10 to 60 min at a controlled temperature of 25 °C ± 2. The isoindole fluorophore was observed to form at approximately 30 min and remained stable for an additional 20 min. However, increasing the reaction temperature above room temperature led to a reduction in RFI of the isoindole product, due to thermal degradation. Consequently, a reaction time of 30 min at room temperature was established as the optimal condition, as presented in Fig. 8.

### Experimental design

**Fig. 9:**
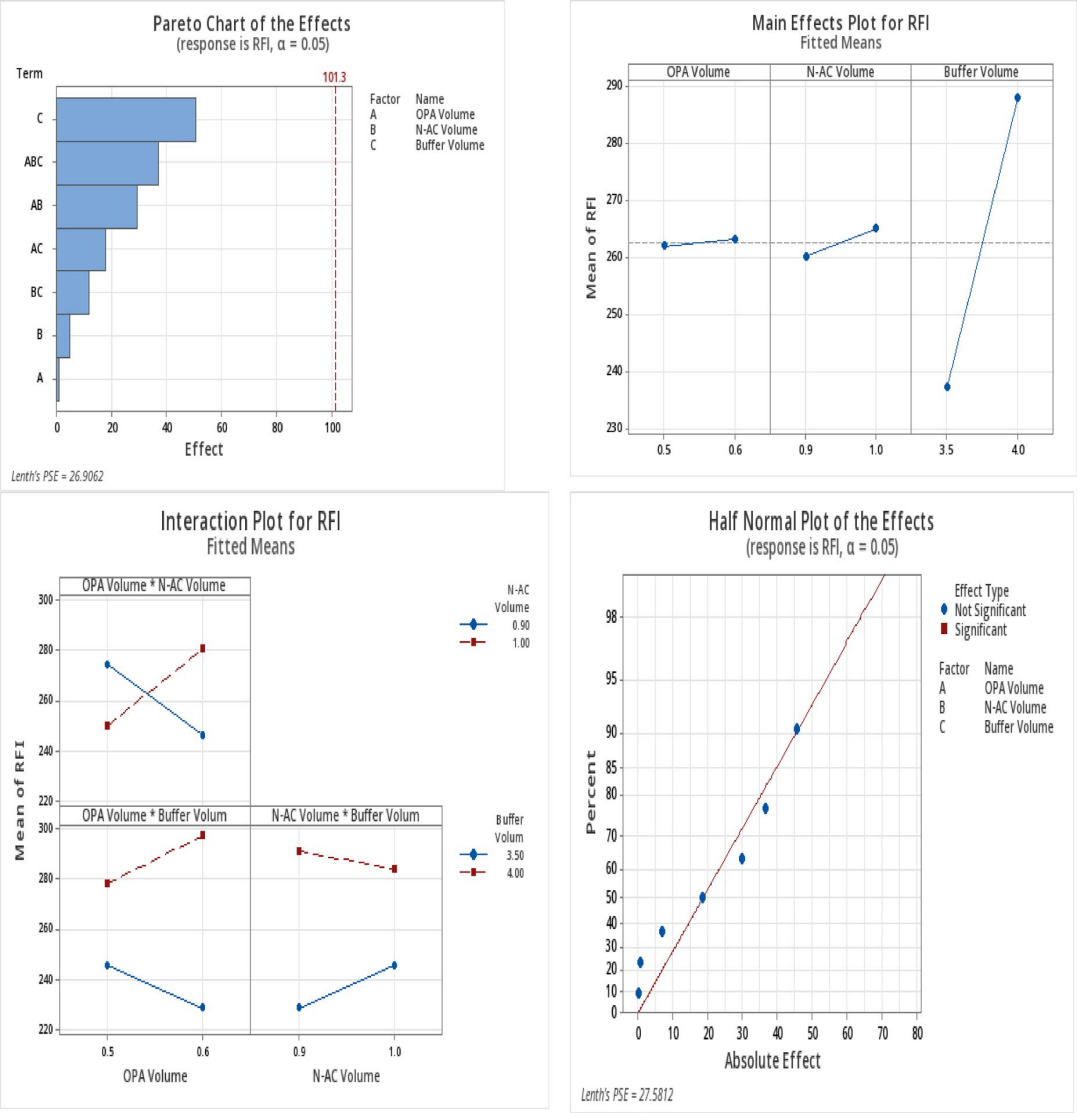
2^3^Full factorial design Pareto chart, main effect plot, interaction plot and half normal plot of the effects on RFI at α = 0.05.

Based on preliminary experiments, the buffer volume and the volumes of the reagents (OPA and N-AC) were identified as the most important independent variables influencing the dependent response RFI. A 2^3^ full factorial design (FFD) comprising eight experiments was employed to systematically investigate the effects of these three factors, buffer volume, OPA volume, and N-AC volume, on the RFI of the resulting isoindole fluorophore, as detailed in Table 1. The factor ranges were carefully selected based on prior optimisation studies described in the method optimisation section. Experimental data were analysed using Minitab^®^ software to optimise the RFI and compute the composite desirability (D) via the response optimiser tool. The desirability function ranges from 0 to 1, with values closer to 1 indicating more favourable conditions. High D values obtained for the method confirmed that optimal conditions were achieved, as shown in Table 2. The software-generated optimisation plots (Fig. 9) suggested that the optimal conditions included 4 mL of borate buffer, pH 11, 600 µL of OPA and 1000 µL of N-AC.

**Table 1:** 2^3^ full factorial design for spectrofluorimetric determination of CTN by derivatisation using OPA/N-AC.

Design Order				Experimental factorial design			Dependent response
Std Order	Run Order	Centre Pt	Blocks	OPA volume (mL)	N-AC volume (mL)	Buffer volume (mL)	RFI
2	1	1	1	0.6	0.9	3.5	224.30
4	2	1	1	0.6	1.0	3.5	233.50
5	3	1	1	0.5	0.9	4.0	315.17
7	4	1	1	0.5	1.0	4.0	241.74
6	5	1	1	0.6	0.9	4.0	267.89
3	6	1	1	0.5	1.0	3.5	258.00
8	7	1	1	0.6	1.0	4.0	327.17
1	8	1	1	0.5	0.9	3.5	233.40

**Table 2:** Response optimisation of a 2^3^ full factorial design for spectrofluorimetric determination of CTN.

Parameters								
	Goal	Lower	Target	Upper	Weight	Import	Predicted Response	Individual Desirability, (d)
**RFI**	Maximise	224.3	327.17	-	1	1	327.17	1.0
**Optimum conditions**: Buffer volume = 4.0 mL OPA volume = 0.6 mL N-AC Volume 1.0 ml							**Composite desirability (D)** = 1.0	

### Method validation

The analytical procedure was validated according to the International Council for Harmonisation (ICH) guidelines^39^. The validation parameters assessed included linearity, range, limit of detection (LOD), limit of quantification (LOQ), accuracy, and precision.

**Table 3:** Validation data for the proposed method for the determination of CTN.

Parameter	Value
Excitation wavelength (λ_ex_)	341 nm
Emission wavelength (λ_em_)	425 nm
Linear Dynamic Range	50.0-300.0 ng/mL
Limit of detection (LOD)	6.4 ng/mL
Limit of quantification (LOQ)	19.5 ng/mL
Correlation coefficient (R^2^)	0.9942
Slope	1.4158
intercept	264.01
Standard deviation of the slope (S_b_)	0.511
Error %	0.194
RSD %	0.512

**Fig. 10:**
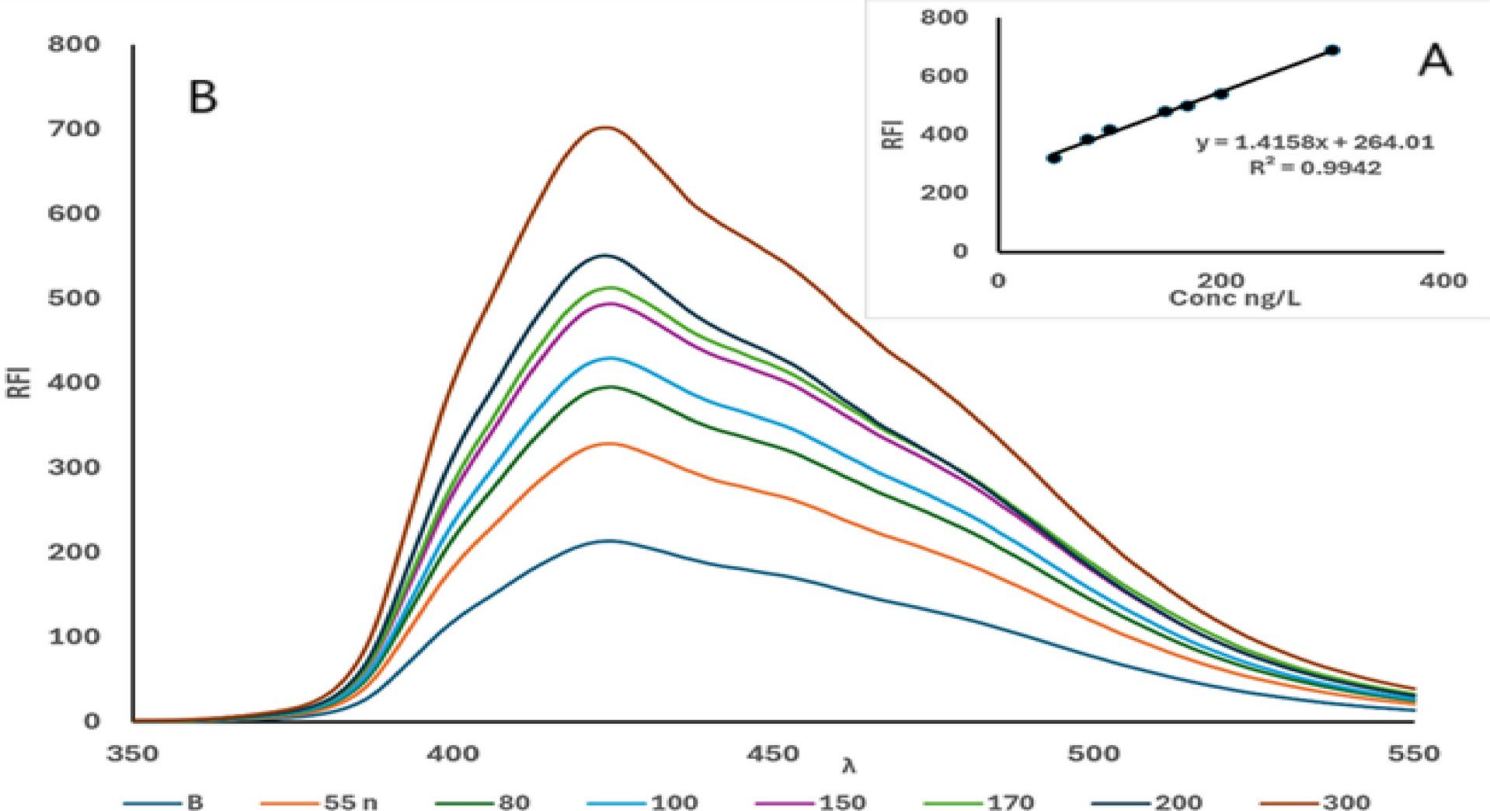
**(A)** Calibration curve for the determination of CTN using the suggested spectrofluorimetric method. **(B)** Emission fluorescence spectra of the reaction product of CTN (50- 300ng/ml) with OPA/N-AC were recorded after excitation at 341 nm.

### Linearity and range

Based on the optimised experimental factors, a linear relationship was established between RFI and the concentration of CTN within the range of 50–300 ng/mL, as illustrated in Fig. 10A and B. The corresponding regression parameters and calibration equation are summarised in Table 3. The regression analysis demonstrated excellent linearity, with a coefficient of determination (*R²*) of 0.9942, indicating the reliability of the approach for quantitative analysis within the specified range.

### LOD and LOQ

The limits of detection (LOD) and quantitation (LOQ) for the proposed approach were calculated using the standard equations: LOQ = 10 × ơ/S and LOD = 3.3 × LOQ, where ơ represents the standard deviation of the intercept and S is the slope of the calibration curve. The data required for these calculations were obtained from the calibration plot. The method demonstrated high sensitivity, as reflected by the calculated LOD and LOQ values of 6.4 ng/mL and 19.5 ng/mL, respectively.

### Precision and accuracy

To assess the accuracy of the developed approach, triplicate measurements were performed at concentrations of 100, 150, and 200 ng/ml, as part of the linearity evaluation. The mean recovery percentage was determined for every tested concentration. The method exhibited strong accuracy, as reflected by consistently high recovery values and standard deviations remaining below 2%.

Repeatability of the approach was evaluated by examining the precision of the suggested method. This involved analysing three replicates at each tested concentration level within a single day (intra-day precision) and across three consecutive days (inter-day precision). Such analysis allows for a comprehensive evaluation of the method’s precision and overall reproducibility. As presented in Table 4, the calculated relative standard deviation (%RSD) values remained below 2%, confirming that the method demonstrates both high precision and reproducibility.

**Table 4:** Accuracy and precision data for the proposed method for analysing the CTN drug.

Parameter	Intra-day precision	Inter-day precision
Concentration ng/mL	100 150 200	100 150 200
%Recovery	99.91 100.43 99.64	99.47 100.78 99.48
±SD	0.76 0.68 0.72	0.89 0.47 0.41
%RSD	0.76 0.68 0.73	0.89 0.47 0.41
%E	0.44 0.39 0.42	0.52 0.27 0.24

#### Application in a pharmaceutical dosage form

The established approach was effectively utilised for the quantitative analysis of CTN in Fortamind ^®^ oral drop. It demonstrated high specificity, with no detectable interference from common excipients, and the procedure required no prior separation, highlighting its simplicity and suitability for routine application. To further confirm the method’s accuracy, the standard addition approach was employed, producing acceptable recovery values as detailed in Table 5. Moreover, the performance of the optimised method was statistically compared to a previously reported spectrofluorimetric technique^25^ using both Student’s *t*-test and the F-test for variance. As summarised in Table 6, the statistical analysis revealed no significant differences between the two approaches, affirming the precision and reliability of the proposed procedure.

**Table 5:** Recovery study for determining CTN in its pharmaceutical dosage form by the standard addition technique.

Parameters	Taken (ng/ml)	Standard addition (ng/ml)	Found (ng/ml)	Mean recovery (%) *
Fortamind ^®^ oral drop (100 mg/ml)	100	50	151.1	100.73
		100	198.9	99.45
		150	252.3	100.93
Mean ± RSD%				100.37 ± 0.69

^*^Each result is an average of three determinations.

**Table 6:** Suggested and reported approaches utilised for the determination of CTN spectrofluorimetry.

Proposed method				Reference method ^25^
Parameters	Taken (ng/ml)	Found (ng/ml)	Mean Recovery %	Recovery %
Fortamind ^®^ 100 mg/mL Oral drops	150	151.9	101.33	
	200	199.2	99.59	
	250	251.7	100.66	
Mean ± RSD%			100.53 ± 0.68	100.29 ± 0.97
Student *t*-test				0.37 (2.57) **
F-test				1.05 (9.55) **

**The value in brackets is the theoretical one at *P* = 0.05.

### Application in synthetic wastewater

Synthetic wastewater offers several benefits, particularly when real samples are difficult to obtain. It reduces the risk of exposure to harmful chemical waste found in actual wastewater and is generally free of unpleasant odour^40^. This type of sample was utilised to assess CTN levels through the standard addition approach. A distinct aliquot of synthetic wastewater was supplemented with three varying concentrations of CTN from the working stock solution. This procedure not only mimics the complexity of real environmental matrices but also enables the rectification of any potential matrix effects.The data shown in Table 7 confirms the method’s effectiveness in detecting the target compound.

**Table 7:** Suggested approach used for the determination of CTN in wastewater.

Parameters	Taken (ng/ml)	Standard addition (ng/ml)	Found (ng/ml)	Mean recovery (%)*
Conc. Of CTN in wastewater	75	50	122.65	98.12
		100	171.39	97.94
		150	220.60	98.04
Mean ± RSD%				98.04 ± 0.67

^*^Each result is an average of three determinations.

#### Green assessment

A comparative evaluation of existing and newly developed methods for CTN determination in analytical chemistry was performed using the RGB model-based framework^41–46^ (Table 8). Published techniques, including spectrophotometry, spectrofluorimetry, RP-HPLC, and LC-MS, were assessed alongside the proposed method using the RGB and WAC scoring systems. The red model-based evaluation focused on validation efficiency. The reported spectrofluorimetric method, which demonstrated nanogram-level sensitivity but was restricted to CTN estimation in pharmaceutical dosage forms, achieved a score of 95/100 (Table 8). In comparison, published UV-spectrophotometry, RP-HPLC, and LC-MS methods obtained scores of 85,80, and 70, whereas the proposed method attained a perfect score of 100. This superior performance is attributed to its nanogram-level sensitivity, suitability for analysis of marketed CTN formulations, implementation of the AQbD approach, excellent recovery percentages, and broad applicability.

The green model-based evaluation of analytical methods was performed using the AGREE calculator, NEMI standards, and the ESA tool. However, literature suggests that NEMI standards provide the least reliable measure of greenness in analytical assessments. Therefore, AGREE scores were primarily considered for calculating the green model-based score^47,48^. This scoring system emphasized the environmental sustainability of the methods. Based on the AGREE calculator, published methods (spectrophotometry, spectrofluorimetry, HPLC, and LC-MS) achieved scores of 75, 71, 65, and 57, respectively, while the proposed method obtained a score of 71. This outcome reflects its reliance on environmentally benign solvents, minimal organic waste generation, and reduced energy consumption in CTN estimation. For time, cost-efficiency, and user-friendliness, the blue model-based assessment was conducted. The published spectrophotometric, spectrofluorimetric, RP-HPLC, and LC-MS methods obtained scores of 100, 100, 80, and 80, respectively. In contrast, the proposed method earned a perfect score of 100, reflecting its economical, rapid, user-friendly, and straightforward design for CTN determination.

The overall WAC score, derived from the average of the RGB model-based evaluations, was 87, 89, and 75.69 for the published spectrophotometric, spectrofluorimetric, RP-HPLC, and LC-MS methods, respectively. The proposed method, however, achieved a higher score of 90, highlighting its combined strengths in validation efficiency, environmental compatibility, speed, cost-effectiveness, and ease of application for CTN estimation.

**Table 8:** Principles of white and green analytical chemistry in the assessment of whiteness and greenness profiles of developed and published methods for CTN estimation.

Principles of White and Green Analytical Chemistry	Method 1: Spectrophotometric method ^6^	Method 2: Spectrofluorimetric method ^25^	Method 4: RP-HPLC^17^	Method 5: LC/MS ^21^	Method 6: Proposed spectrofluorimetric
**Red Model-based assessment of analytical methods**					
R1-Scope and applications	Pure form and pharmaceutical dosage form	Pure form and pharmaceutical dosage form	Pure form and pharmaceutical dosage form	In a pharmaceutical dosage form	In a pure pharmaceutical dosage form and wastewater
R2-Linearity LOD and LOQ	-5–55 µg/ml -LOD 0.28 µg/ml -LOQ 0.85 µg/ml	-300–3000 ng/mL -LOD 291.0 ng/mL -LOQ 93.89 ng/mL	**-**10–60 µg/ml **-**LOD 0.48 µg/ml -LOQ 1.5 µg/ml	125–375 µg/ml	50.0–300.0 ng/mL -LOD 6.4 ng/mL -LOQ 19.5 ng/mL
R3-Precision	As per ICH Q2 (R1)	As per ICH Q2 (R1)	As per ICH Q2 (R1)	As per ICH Q2 (R1)	As per ICH Q2 (R1)
R4-Accuracy	Recovery is 99.79%	Recovery is 100.32%	As per ICH Q2 (R1)	Recovery is 98.4%	Recovery is 99.95%
Red Model Score	**85**	**95**	**80**	**70**	**100**
**Green Model-based assessment of analytical methods**					
NEMI	Less hazardous &corrosive solvents and less waste organic generation	Less hazardous &corrosive solvents and less waste organic generation	More hazardous & corrosive solvents and less waste organic generation	More hazardous & corrosive solvents and less waste organic generation	Less hazardous &corrosive solvents and less waste organic generation
AGREE Score					
Eco-scale	**95**	**88**	**88**	**74**	**82**
Green model score	**75**	**71**	**65**	**57**	**71**
**Blue Model-based assessment of analytical methods**					
B1-Cost of analysis	Low cost	Low cost	High cost	High cost	Low cost
B2-Time efficiency	Less time required	Less time required	Less time required	More time required	Less time required
B3-Sample analysis requirements	Less than 3 steps of analysis	Less than 3 steps of analysis	Less than 3 steps of analysis	Less than 3 steps of analysis	Less than 3 steps of analysis
B4-user friendliness	More user-friendly	More user-friendly	Less user-friendly	Less user-friendly	More user-friendly
Blue model score	**100**	**100**	**80**	**80**	**100**
**White analytical Chemistry (WAC)-based assessment of analytical methods**					
WAC score = Average of RGB models’ score	Average of (85 + 75 + 100) = 87	Average of (95 + 71 + 100) = 89	Average of (80 + 65 + 80) = 75	Average of (70 + 57 + 80) = 69	Average of (100 + 71 + 100) = 90
Whiteness and Greenness status	Excellent white and green method	Excellent white and green method	Good white and green method	Good white and green method	Excellent white and green method

## Conclusion

This study developed and validated a highly sensitive spectrofluorimetric approach for the determination of CTN, demonstrating excellent performance in analysing pharmaceutical dosage forms without interference from common excipients. This makes the approach particularly suitable for routine use in quality control laboratories. Its high sensitivity also allows for effective application in wastewater analysis, supporting environmental monitoring efforts.

Compared to more complex and costly analytical techniques such as gas chromatography (GC) and high-performance liquid chromatography (HPLC), the spectrofluorimetric approach offers a simpler, more cost-effective, and eco-friendly alternative. The approach employs green solvents, selected for their widespread availability, lower cost, and reduced environmental impact compared to other solvents. The incorporation of green solvents enhances the environmental sustainability of the approach, reinforcing its role as a competitive and responsible solution for pharmaceutical quality control and environmental analysis.

## Data Availability

All data generated or analysed during this study are included in this published article.
